# Selective hydrogen bonding enables solar-driven amine removal from carbon capture systems

**DOI:** 10.1039/d6sc05691a

**Published:** 2026-09-24

**Authors:** Yumeng Xiao, Xinru Yang, Pengyang Liu, Xiangqian Song, Lidong Wang, Ruimin Zhao, Tony D. James, Meng Li

**Affiliations:** a Hebei Key Lab of Power Plant Flue Gas Multi-Pollutants Control, Department of Environmental Science and Engineering, North China Electric Power University Baoding 071003 P. R. China wld@ncepu.edu.cn mli@ncepu.edu.cn; b MOE Key Lab Resources and Environmental System Optimization, College of Environmental Science and Engineering, North China Electric Power University Beijing 102206 P. R. China; c Department of Chemistry, University of Bath Bath BA2 7AY UK t.d.james@bath.ac.uk; d School of Chemistry and Chemical Engineering, Henan Normal University Xinxiang 453007 P. R. China

## Abstract

Amine-based CO_2_ capture suffers from volatile amine escape, causing atmospheric emissions, while the widely used water scrubbers for emission control produce large amounts of alkanolamine-containing wastewater (ie monoethanolamine (MEA)). However, solar-driven purification, a promising low-energy treatment for such wastewater, is challenged by amine co-evaporation. Herein, a bi-functional graphene oxide based polyvinyl alcohol aerogel (GPA) was developed for synergistic MEA capture and water evaporation with long-term stability, achieving 99.5% MEA removal and an evaporation rate of 1.36 kg m^−2^ h^−1^ under 1 sun illumination. Combined DFT and MD simulations reveal that hydroxyl (–OH) from the polyvinyl alcohol and carboxyl (–COOH) from graphene oxide synergistically establish selective hydrogen bonding interactions with MEA, where –COOH exhibited exceptionally long hydrogen-bond lifetimes (6.1 ps), thereby immobilizing MEA whilst also allowing rapid water diffusion. GPA maintains >95% MEA removal over 20 consecutive days and under extreme pH conditions. Life cycle assessment indicates that GPA has the lowest environmental impact among comparable systems, accounting for only 6% of global warming impact. This work resolves the amine co-evaporation bottleneck and provides a sustainable pathway to manage alkanolamine-laden wastewater from carbon capture systems.

## Abstract 2

A bifunctional aerogel with selective hydrogen-bonding interactions enables efficient solar-driven amine removal and water recovery from carbon capture wastewater.

## Graphical abstract

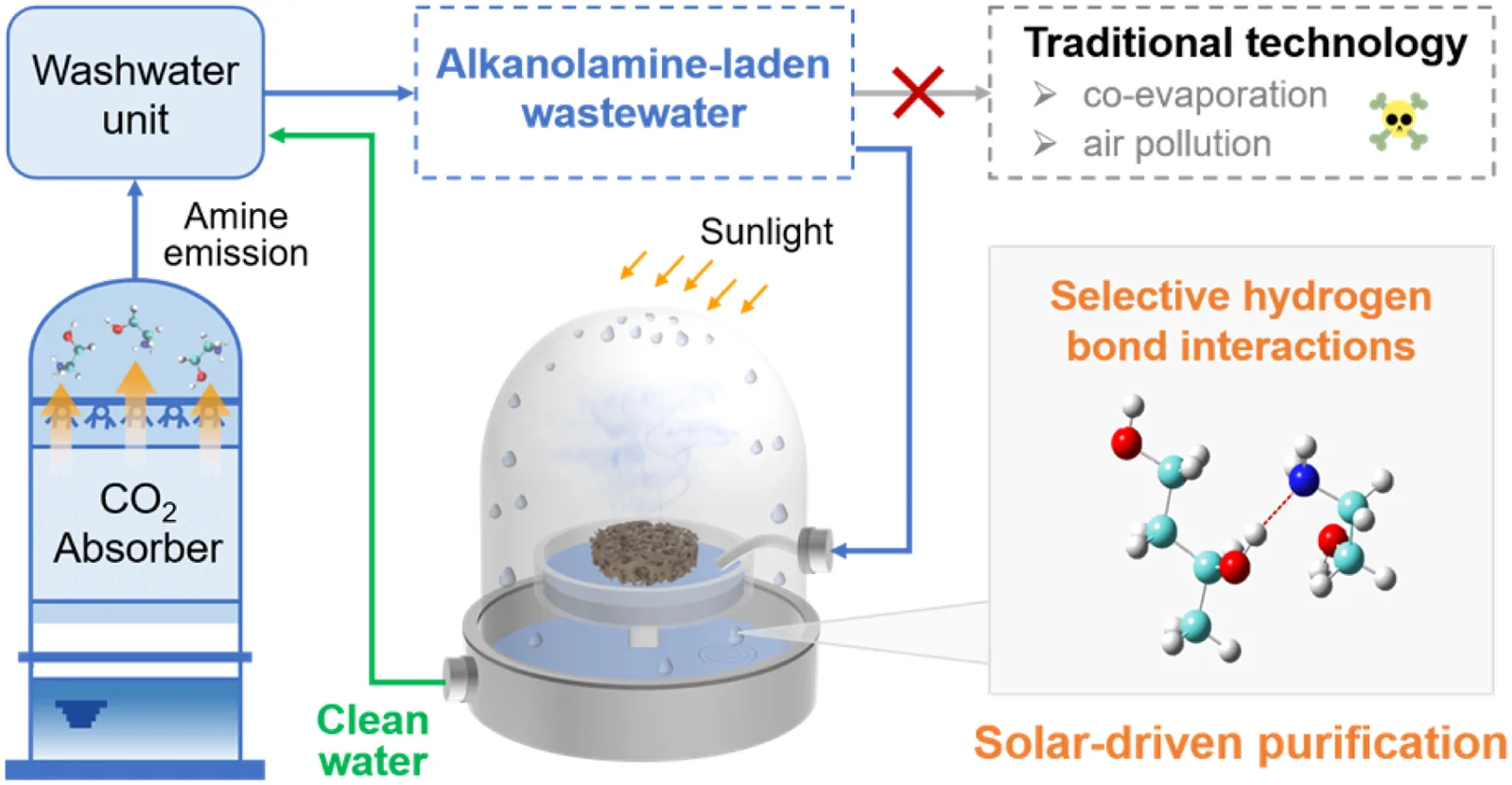

## 1. Introduction

Carbon capture is indispensable for mitigating CO_2_ emissions from existing power and industrial plants.^1–3^ Among available technologies, alkanolamine-based absorption remains the most widely adopted due to its high CO_2_ uptake capacity and favourable kinetics.^4,5^ However, the high organic content results in significant amine emission, which is commonly addressed using water scrubbing/washing towers.^6–8^ While effective washing control demands substantial freshwater consumption and results in the production of alkanolamine-laden wastewater.^7^ Direct discharge of such wastewater poses potential risks to aquatic ecosystems and human health, yet alkanolamines as an emerging class of pollutants remain largely underexplored.^9^ Conventional purification routes, including chemical precipitation and membrane separation, remain constrained by high energy consumption, high chemical demand, and the risk of secondary pollution.^10^ Consequently, there is a pressing need for advanced treatment strategies that can specifically and sustainably decontaminate amine-rich wastewater.

Solar-driven water evaporation offers a low-carbon, self-sustaining alternative that harnesses abundant solar energy to produce clean water from contaminated sources, making it attractive for amine-rich wastewater treatment.^11–14^ However, its practical application is impeded by the intrinsic volatility of alkanolamines, which co-evaporate with water vapor, leading to the release of alkanolamines into the atmosphere.^15,16^ This co-evaporation issue has been largely overlooked in existing solar evaporator designs, which typically prioritize evaporation rate over selective retention of volatile organic compounds (VOCs). Therefore, three prerequisites for efficiently intercepting alkanolamines during solar-driven water remediation are essential: (1) construct evaporators exhibiting strong, specific molecular interactions that can selectively capture alkanolamines while allowing water to pass through; (2) develop evaporators able to sustain superior photothermal conversion performance and promote water diffusion; (3) develop evaporators exhibiting long term stability and sustainability to enhance the potential of practical application.

**Fig. 1:**
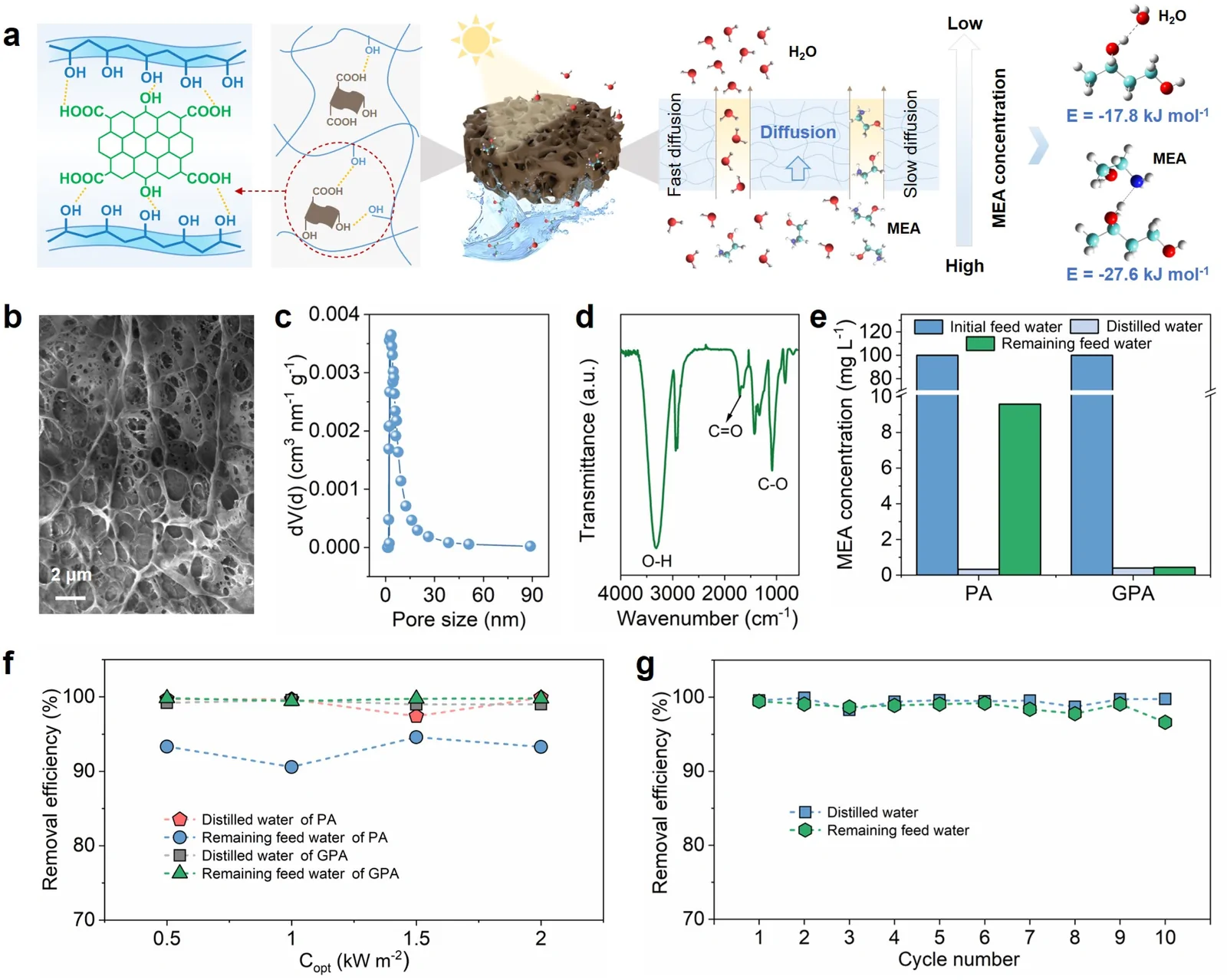
Characterization and investigation of MEA removal performance. (a) Schematic illustration of GPA for MEA removal. (b) SEM image of GPA. (c) Pore size distribution of GPA. (d) FTIR spectrum showing the oxygen-containing functional groups in GPA. (e) Measured concentrations of MEA in distilled water and remaining feed water for different systems under 1 sun. It should be noted that the initial concentration of MEA is 100 mg L^−1^. (f) Relationship between MEA removal efficiency in different systems under different intensities of light and solar irradiation. (g) Cycling performance of GPA under 1 sun.

With this research, we constructed a facile bifunctional graphene oxide (GO)-polyvinyl alcohol (PVA) aerogel evaporator (GPA) to produce clean water while suppressing alkanolamine volatilization through selective hydrogen bonding. Using monoethanolamine (MEA) as the model pollutant, GPA achieves 99.5% MEA removal and an evaporation rate of 1.36 kg m^−2^ h^−1^ under 1 sun illumination. Molecular dynamics (MD) simulations and density functional theory (DFT) calculations revealed that GPA forms stronger hydrogen bonds with MEA than with water, primarily through a synergy of PVA and GO. These selective interactions, together with the larger molecular size of MEA, effectively restrict MEA diffusion across GPA while facilitating the transport of water molecules (Fig. 1a). Importantly, GPA maintained high removal efficiency and evaporation performance over prolonged operation and multiple cycles, even under chemically challenging conditions. This study provides a new strategy for sustainable removal of MEA and advances the environmental feasibility of amine-based carbon capture.

## 2. Experimental methods

### 2.1 Fabrication of GO–PVA aerogel (GPA)

GO 300 mg was dispersed into an appropriate amount of DI water and sonicated for 2 h. The precipitate obtained after centrifugation, 100 µL of 50 wt% glutaraldehyde solution and 1 mL of 1.2 M HCl aqueous solution was added to the PVA precursor solution, which was stirred evenly and kept at room temperature for 2 h. The sample was washed with DI water and freeze-dried to obtain GPA. The preparation of PVA precursor solution can be found in SI Section 1.2.

### 2.2 Characterization

The morphology of the samples was analyzed by scanning electron microscopy (SEM, Hitachi-s4800). The absorption spectra were obtained using a UV-vis-NIR spectrometer (Cary 5000). Fourier transform infrared (FTIR) spectra were acquired using a Thermo Electron Nicolet iZ10 spectrometer within the spectral range of 4500–500 cm^−1^.

### 2.3 Solar steam generation and efficiency calculation

The water evaporation experiment was conducted in a 50 mL crystallizing dish filled with 30 mL of primary treatment water. The samples were irradiated under a solar simulator with 100 mW cm^−2^ at room temperature and a humidity of about 50%. The mass changes of the system were measured on an electronic balance, while temperature changes were monitored in real time with an infrared camera. The solar-to-vapor conversion efficiency (*η*) can be calculated by the following equation:^17,18^1
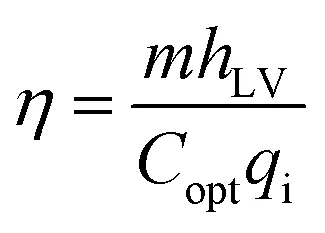
where *η* is solar-to-vapor conversion efficiency; *m* is the mass flux of the steam; *h*_LV_ represents the vaporization enthalpy of the water in the samples, specifically set at 2444 J g^−1^, with no consideration for sensible heat;^19^*C*_opt_ is optical concentration and *q*_i_ is the solar irradiation power on the adsorber surface (100 mW cm^−2^).

### 2.4 Evaluation of alkanolamine removal

The removal performance of the samples was evaluated under simulated solar irradiation (light intensity can be tuned from 100 to 300 mW cm^−2^). Place the sample at the interface of 100 mg L^−1^ MEA solution, a model pollutant found in industrial wastewater from CO_2_ capture systems. After 1 hour of illumination, take a proper amount of distilled water and remaining feed water, and measure the concentration by high performance liquid chromatograph (HPLC, LC-20AT-Shimadzu). The removal efficiency of the samples for alkanolamine can be calculated by the following equation:^20^2
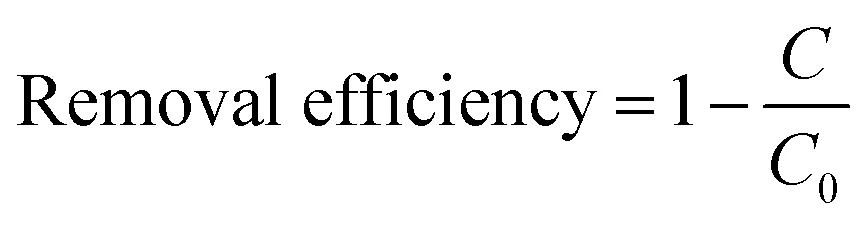
where *C* represents real-time concentration and *C*_0_ represents initial concentration.

For the cycling test, after each cycle, the GPA was rinsed with deionized water and then used in a fresh MEA solution for the subsequent cycle.

### 2.5 Theoretical calculations

The DFT calculations were performed using the Gaussian16 program, and MD simulations were performed using the Gromacs 2025.2 software. Detailed parameters are provided in SI Section 1.4 and 1.5.

### 2.6 Life cycle assessment (LCA)

In order to evaluate the environmental impact of the GPA system and to compare it with other similar technologies, four systems for solar thermal evaporation of volatile organic pollutants were selected. LCA was used to calculate and compare the environmental impact of these four systems according to ISO standard 14040, quantifying their impacts using the Ecoinvent v3 database. In this study, 1 kg of material was the functional unit, excluding construction, excavation, and waste disposal. Inventory analysis of each system is given in Tables S2–S6.

## 3. Results and discussion

### 3.1 Construction of a bifunctional GO–PVA aerogel with high MEA removal efficiency

The fabrication process of GPA is depicted schematically in Fig. S1. GPA was fabricated by dispersing GO precipitate into PVA precursor solution, followed by cross-linking with glutaraldehyde and HCl, and then freeze-drying. As seen in Fig. 1a, the chemical structure of GPA features a cross-linked PVA–GO network abundant in oxygen-containing functional groups. The micromorphological characteristics of GPA can be observed from scanning electron microscopy (SEM) image (Fig. 1b). GPA exhibits a smooth surface with slight wrinkling, suggesting a uniform distribution of GO within the PVA matrix, which can enhance light absorption.^21^ In addition, the pore size of GPA is in the range of 2 to 15 nm (Fig. 1c), and it exhibits exceptional hydrophilicity with a water absorption time of 1.68 s (Fig. S2). These properties are expected to promote efficient pathways for water.^22^ To further verify the chemical composition, Fourier transform infrared (FTIR) spectroscopy of GPA was performed (Fig. 1d). Characteristic peaks at 1088 cm^−1^ and 3324 cm^−1^ are observed, corresponding to C–O and O–H stretching vibrations, respectively. The absorption band at 1701 cm^−1^ is attributed to C=O stretching.^23,24^ These abundant oxygen-containing functional groups can provide active sites for the interaction between GPA and MEA.^25,26^

The MEA removal performance of GPA was evaluated in a solar-driven purification device (Fig. S3). As shown in Fig. 1e, with an initial concentration of 100 mg L^−1^, both materials suppressed MEA volatilization into distilled water (∼0.3 mg L^−1^). However, the remaining feed water contained 9.58 mg L^−1^ of MEA for pure PVA aerogel (PA), whereas GPA left only 0.44 mg L^−1^, achieving a removal efficiency of 99.5%. These results confirm that GPA effectively suppressed MEA from evaporating and simultaneously prevented its concentration build-up in the remaining feed water. To verify whether MEA was degraded, HPLC analysis was performed to qualitatively identify formic acid, a typical degradation product of MEA.^27^ As shown in Fig. S4, the formic acid standard exhibited a characteristic peak at about 3.6 min. In contrast, both the initial MEA solution and the remaining feed water after treatment displayed identical peaks at about 2.9 min, and no peak corresponding to formic acid was detected. These results confirm that MEA was not degraded into formic acid but was mainly removed through adsorption. To further confirm this, the XPS spectrum of GPA–MEA is given in Fig. S5, in which an obvious N signal was observed. In the high-resolution N 1s XPS spectra of GPA-MEA (Fig. S6), the peak cantered at 399.69 eV is indicative of free amine (–NH_2_), and the peak at 401.56 eV is indicative of –NH_3_^+^ groups arising from possible direct acid–base interactions with the carboxylic and hydroxyl groups on GO, confirming the adsorption of MEA.^28^

Moreover, the stability of GPA for treating MEA-contaminated water was examined under various conditions. As demonstrated in Fig. 1f, the removal efficiency of PA fluctuated with increasing irradiation intensity (0.5–2 sun), and remained below that of GPA. GPA maintained over 99% MEA removal efficiency under different irradiation intensities, confirming its robust and thermally stable purification capability. Significantly, GPA retained over 96% MEA removal efficiency during a 10-cycle test under 1 sun irradiation (Fig. 1g). Given that carbon capture wastewater may contain residual heavy metals (*e.g.*, Hg^2+^),^29^ the performance of GPA under complex contamination was evaluated, demonstrating 97% MEA removal and 99.9% Hg^2+^ elimination (Fig. S7).

### 3.2 Selective hydrogen bond-mediated MEA adsorption mechanism

**Fig. 2:**
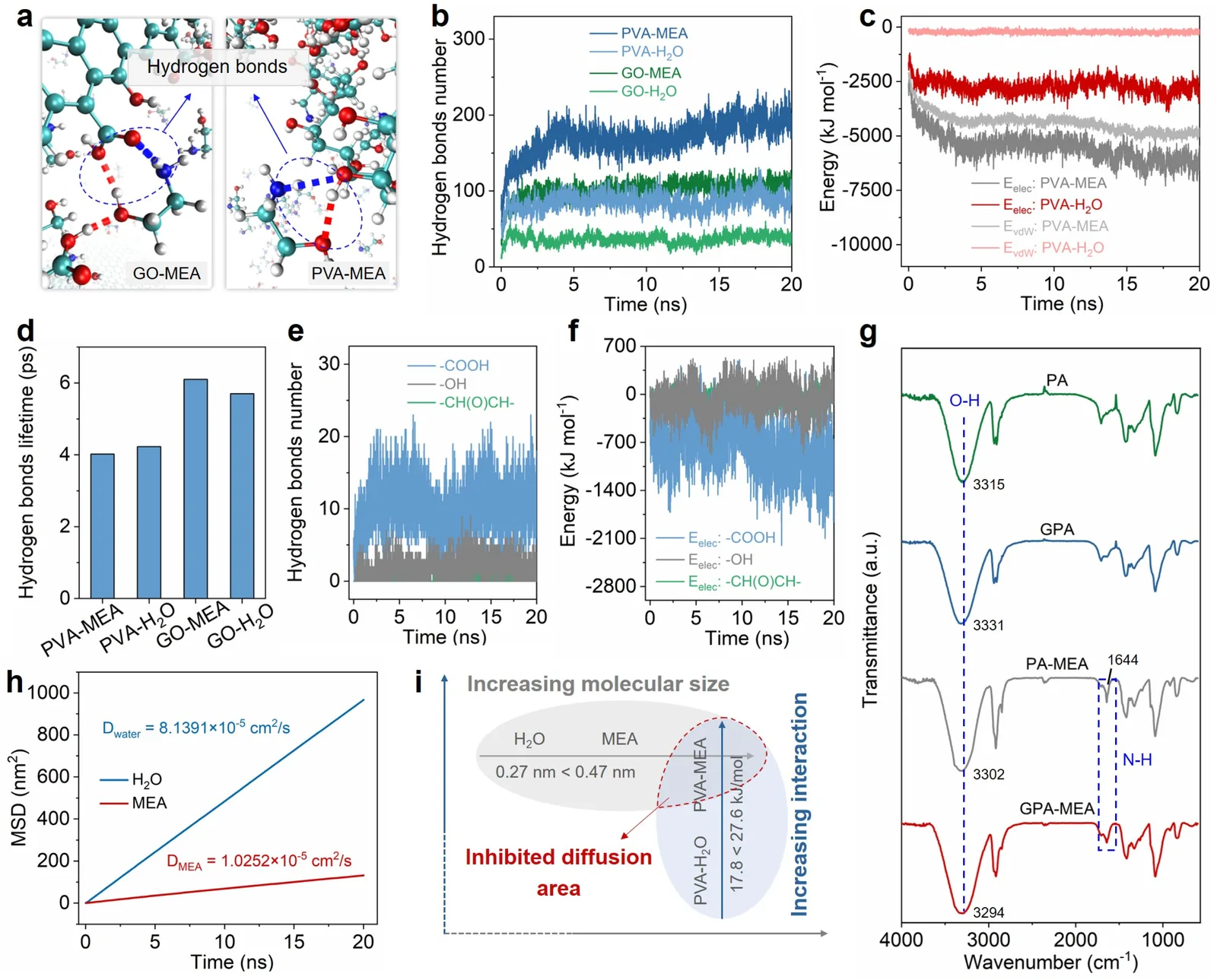
Investigation of selective capture mechanism for MEA enhanced by functional groups. (a) Interaction between MEA molecules and GO, PVA. (b) Number of hydrogen bonds between the four components in the system. (c) Electrostatic and vdW interactions of PVA–MEA and PVA–H_2_O. (d) Lifetime of hydrogen bonds between the four components of the systems. (e) Number of hydrogen bonds between different functional groups and MEA molecules. (f) Electrostatic interactions between different functional groups and MEA molecules. (g) FTIR of PA, GPA, PA–MEA and GPA–MEA. (h) Mean squared displacement (MSD) and diffusion coefficients (*D*) of MEA and water molecules within GPA. (i) Effect of molecule size and interactions on water and MEA diffusion for GPA.

To understand the MEA adsorption mechanism at the molecular level, the differential interaction behaviour of H_2_O and MEA within GPA was evaluated using MD simulations (Fig. S8 and 2a). As shown in Fig. 2b, the number of hydrogen bonds is higher in both PVA–MEA and GO–MEA systems than in PVA–H_2_O and GO–H_2_O systems, indicating that GPA exhibits a greater propensity to form hydrogen bonds with MEA. Where PVA–MEA has the highest number of hydrogen bonds, which is attributed to the abundant –OH groups in PVA. In addition, the interaction energies between the components further explain the selectivity of GPA to MEA. For both PVA and GO, the electrostatic and van der Waals (vdW) interactions with MEA are substantially stronger than those with H_2_O (Fig. 2c and S9), resulting in a markedly higher overall binding strength between MEA and GPA. Hydrogen bond lifetime analysis (Fig. 2d) further reveals that the GO–MEA hydrogen bond exhibits a longer lifetime (6.1 ps) than both PVA–MEA (4.02 ps) and GO–H_2_O (5.7 ps), which is consistent with the stronger overall interaction between GPA and MEA. Among the functional groups of GO, –COOH forms the most hydrogen bonds with MEA (Fig. 2e) and mediates the strongest electrostatic (Fig. 2f) and vdW (Fig. S10) interactions, suggesting that –COOH is a key site leading to selective hydrogen bonding. Therefore, PVA provides abundant –OH that form hydrogen bonds with MEA, while GO offers hydrogen bond sites with enhanced stability and extends hydrogen bond lifetimes primarily *via* –COOH, amplifying the selective immobilization of MEA.

The hydrogen bonding interaction mechanism was further confirmed by FTIR spectra (Fig. 2g). Peaks at 1644 cm^−1^ were assigned to the bending vibration of N–H in MEA, confirming its successful adsorption.^30^ In the spectrum of PA–MEA, the O–H peak exhibited a red shift of 13 cm^−1^ relative to that of pure PA. In contrast, the O–H peak in GPA-MEA was observed at 3294 cm^−1^, corresponding to a more pronounced red shift of 37 cm^−1^. This indicates that the introduction of GO enhances the hydrogen bonding interaction between MEA and the composite system.^31^ These observations are consistent with the results of MD simulations.

The selective hydrogen bonding mechanism established above directly governs the differential transport of H_2_O and MEA in GPA. This transport can be described as a selective solution-diffusion process: molecules first dissolve at the surface, reach equilibrium, and then diffuse through GPA, which is closely related to the interaction between the molecules and GPA and determines the rate of mass transfer.^23,32,33^ The diffusion coefficient (*D*) calculations (Fig. 2h) reveal that *D*_water_ (8.1391 × 10^−5^ cm^2^ s^−1^) is higher than *D*_MEA_ (1.0252 × 10^−5^ cm^2^ s^−1^), indicating that MEA diffusion is significantly restricted whereas H_2_O molecules exhibit higher mobility. This result is attributed to the stronger hydrogen bonding interaction between GPA and MEA (Fig. S11). The larger molecular size and stronger interactions with the diffusion medium generally diffuse at a slower rate (Fig. 2i).^32^ As a result, the stronger hydrogen bonds between GPA and MEA arising from the synergy of PVA and GO effectively restrict MEA diffusion, enabling its selective separation from water.

### 3.3 Excellent and stable solar-driven evaporation performance

**Fig. 3:**
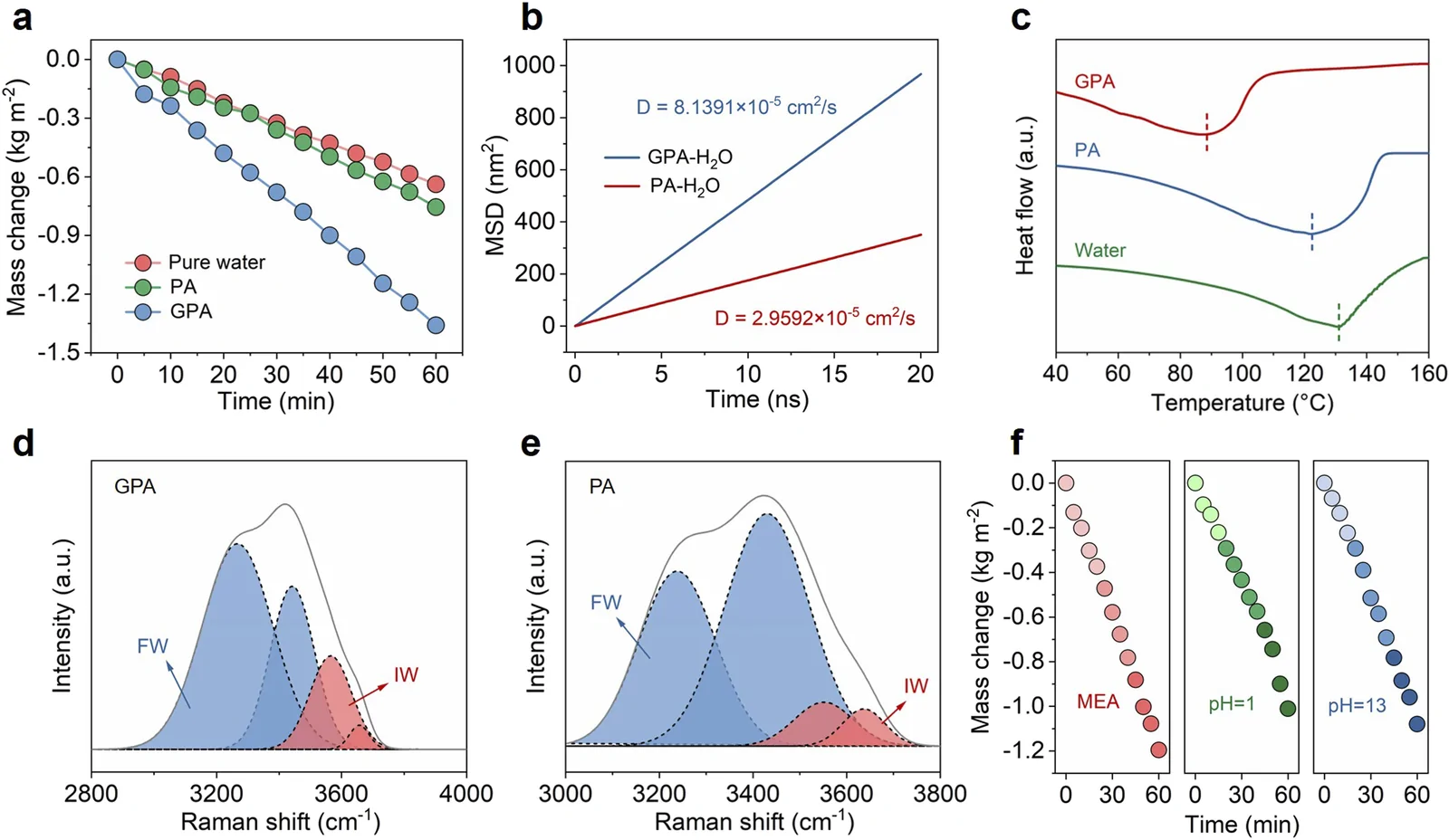
Analysis of evaporation performance and GO-promoted evaporation process. (a) Mass change for different systems under 1 sun. (b) MSD and diffusion coefficients (*D*) of water molecules within GPA and PA. (c) DSC evaluation of water evaporation temperature. Raman spectroscopy showing the fitting peaks representing intermediate water (IW) and free water (FW) in (d) GPA and (e) PA. (f) Variation of evaporation rates using GPA, with treatment under strong acid, alkali and without treatment respectively.

The evaporation performance of GPA was evaluated under simulated sunlight. Efficient broadband solar absorption and photothermal conversion are critical for solar-driven evaporation.^34–36^ GPA exhibited effective light absorption (Fig. S12) due to the light-trapping properties of GO.^37,38^ Under 1 sun, the surface temperature of GPA increased rapidly from 26.8 to 53.4 °C within 30 min and then stabilized (Fig. S13 and S14). As shown in Fig. 3a, GPA exhibited an evaporation rate of 1.36 kg m^−2^ h^−1^, significantly higher than that of PA (0.76 kg m^−2^ h^−1^) and comparable to those of recently reported aerogel-based evaporators (Table S1).^39–43^ With dark evaporation rates of 0.209 and 0.161 kg m^−2^ h^−1^ for GPA and PA, respectively, GPA exhibits a solar-to-vapor conversion efficiency of 79%, compared with 41% for PA (Fig. S15). To elucidate the enhancement mechanism, MD simulations were performed (Fig S16). Fig. 3b shows that *D*_GPA–water_ (8.1391 × 10^−5^ cm^2^ s^−1^) is higher than *D*_PA–water_ (2.9592 × 10^−5^ cm^2^ s^−1^), indicating that GO incorporation enhances water mobility. In hydrated polymers, water exists as bound water (BW), intermediate water (IW), and free water (FW), where the IW region with weaker bonding energy is easier to evaporate.^44^ Differential scanning calorimetry (DSC) verified that GPA exhibited a lower evaporation temperature than pure water and PA (Fig. 3c). Raman O–H stretching spectra (Fig. 3d and e) show peaks at 3233 and 3401 cm^−1^ for FW, and at 3514 and 3630 cm^−1^ for IW.^45^ The IW/FW ratios for PA and GPA were determined to be 0.126 and 0.365, respectively. This confirmed that GO increases the proportion of IW, promoting evaporation at lower energy. Thus, GO plays a crucial role in enhancing evaporation performance.

Furthermore, the stability of GPA under more challenging conditions was evaluated. The evaporation rate and surface temperature both increased with rising irradiation power density (Fig. S17 and S18), demonstrating GPA's effective photothermal response under varying light intensities. Notably, GPA demonstrated a stable evaporation rate of 1.30 kg m^−2^ h^−1^ over 10 cycles (Fig. S19). The evaporation performance was also evaluated in MEA solutions under strongly acidic and alkaline conditions. As shown in Fig. 3f, the water evaporation rate remained similar to that in the untreated MEA solution, indicating the high acid/alkali resistance of GPA. In addition, the presence of Hg^2+^ in the MEA solution resulted in a slight decrease in the evaporation rate to 1.2 kg m^−2^ h^−1^ (Fig. S20), confirming that the co-existing heavy metal ion does not significantly impair the evaporation performance. These results demonstrate that GPA exhibits robust stability, making it a promising candidate for treating industrial carbon capture wastewater.

### 3.4 Outstanding adaptability and long-term stability

**Fig. 4:**
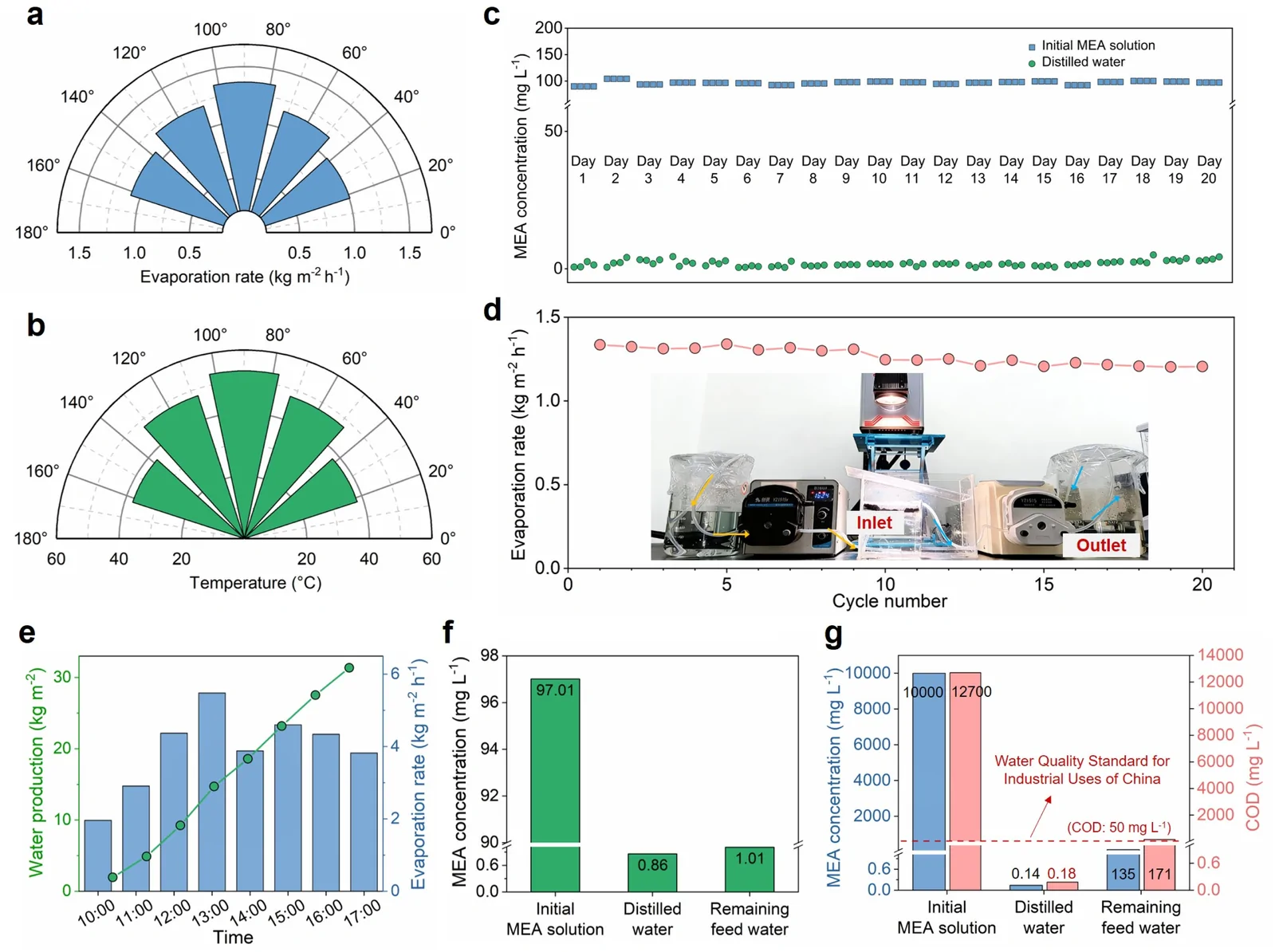
Feasibility of GPA solar-driven purification. (a) Evaporation rate and (b) maximum temperature under different incident light angles. (c) MEA concentration of the distilled water during a continuous experiment, with GPA for water purification under 1 sun (initial concentration was 100 mg L^−1^). (d) Evaporation rate measurement of GPA during 20-days of continuous evaporation, with solar irradiation for 8 hours at 1 kW m^−2^ per day. (e) Variation of evaporation rate during a day. (f) Effect of GPA on MEA removal under outdoor conditions. (g) The test of MEA removal performance at high concentration.

To evaluate the adaptability of GPA to different solar incident angles, evaporation experiments were conducted under five incident angles: 150°, 120°, 90°, 60° and 30° (Fig. 4a), and the corresponding surface temperatures were recorded (Fig. 4b).^46^

Compared with vertical illumination, both evaporation rate and the maximum surface temperature showed only slight fluctuations, confirming reliable operation under various incident angles. The long-term durability of GPA was further evaluated in a 20-day continuous experiment using MEA solution (100 mg L^−1^) under 1 sun irradiation. The system was operated for 8 h per day at a flow rate of 2.5 mL min^−1^, and the distilled water was collected every 2 h for analysis.^47^ The distilled water exhibited stable MEA removal (over 95%) during the 20-day test (Fig. 4c), and the evaporation rate remained steady with only a slight decrease (Fig. 4d), indicating the excellent long-term performance and durability.

To further assess the practical evaporation performance of the evaporator, an outdoor experiment was conducted on August 12, 2024, in Baoding, China (Longitude: 115.46481 E, Latitude: 38.873891 N, temperature of around 33 °C with 68% humidity, and level 1 Northeast wind). The solar intensity, ambient temperature, and water evaporation rate during the outdoor experiments were monitored (Fig. S21 and 4e). Under natural sunlight, the system achieved a maximum evaporation rate of 5.48 kg m^−2^ h^−1^, which is substantially higher than that measured under standard 1 sun irradiation in the laboratory. The high 5.48 kg m^−2^ h^−1^ rate of evaporation was attributed to several factors, including the incident angle of natural sunlight, which enables both the top surface and sidewalls of the evaporator to participate in vapor generation. In addition, the surface of the evaporator is effectively cooled by air flow in the outdoor setting, with the combination of high temperature and airflow enhancing the evaporation efficiency. In addition, the outdoor light intensity was higher than 1 sun during a sunny summer day.^19,48,49^ Crucially, the MEA removal efficiency remained exceptionally high at 99% (Fig. 4f), confirming the robustness of the selective hydrogen bond-mediated capture in real-world conditions. To assess the of GPA, a large-area evaporator (60 cm × 40 cm) was fabricated. Then, an evaporation experiment was conducted with MEA solution at a high concentration of 10 000 ppm (Fig. S22). Fig. 4g shows that the MEA removal efficiency reached over 98.5%, confirming that the scalable fabrication process does not compromise the purification performance. Moreover, the MEA concentration was converted into chemical oxygen demand (COD) at 1.27 g COD per g MEA using an appropriate COD conversion factor. The COD of distilled water was decreased to 0.18 mg L^−1^, which meets the COD concentration requirement of Water Quality Standard for Industrial Uses of China (50 mg L^−1^, GB/T 19923-2024). These findings demonstrate the potential of GPA for practical industrial applications.

### 3.5 Beyond comparable systems with lower costs and carbon emissions

**Fig. 5:**
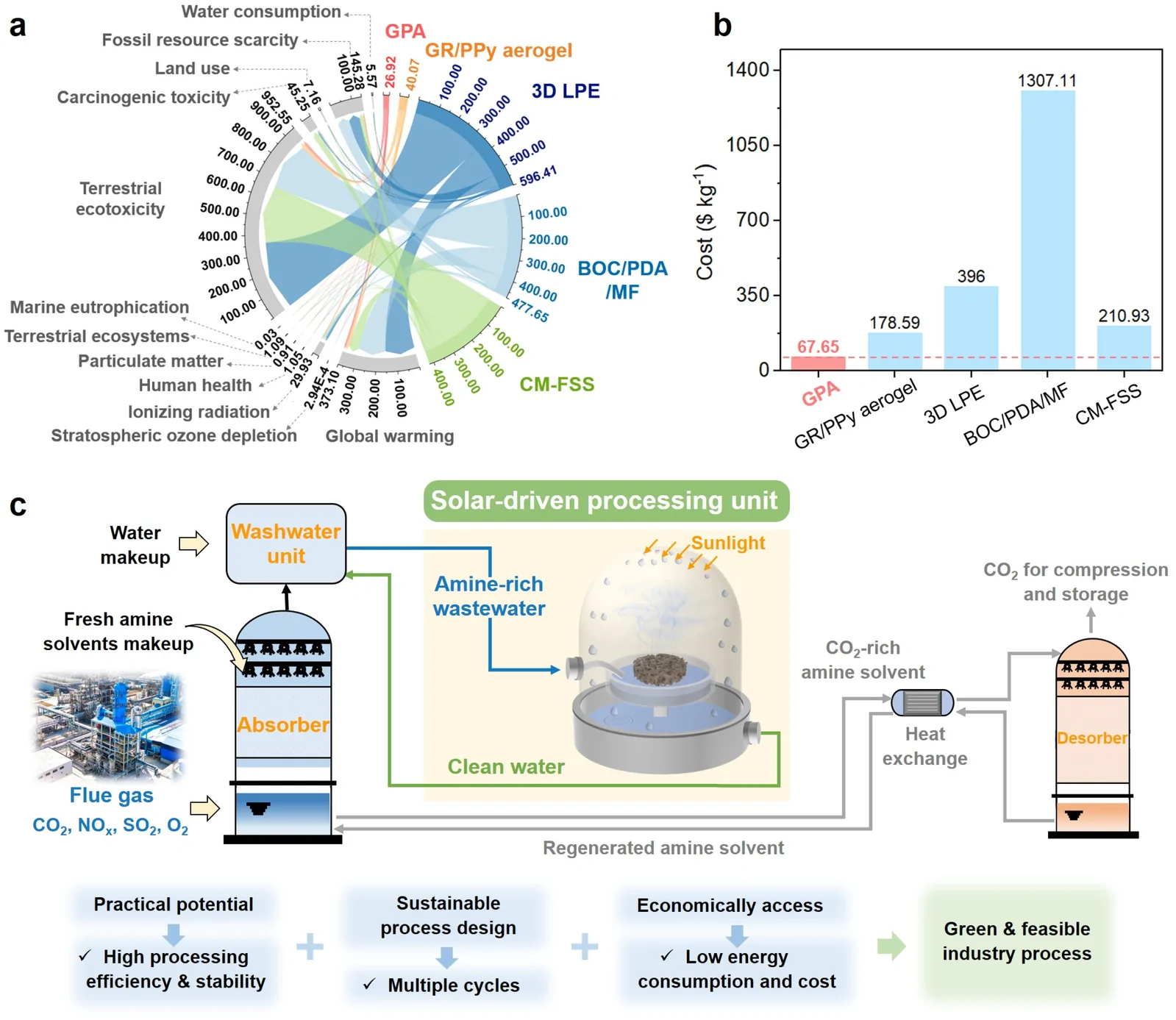
Environmental impact assessment and cost analysis. (a) Comparison of the sum of environmental impacts of five purification systems evaluated. (b) Comparison of material costs for five purification systems. (c) Schematic illustration of next-generation carbon capture systems for amine-rich wastewater control strategy.

To evaluate the environmental impact of the GPA system, LCA was conducted and compared with four specific purification technologies, including: (1) an integrated reduced graphene oxide/polypyrrole (GR/PPy) hybrid aerogel,^20^ (2) a light-permeable solar evaporator with three-dimensional photocatalytic sites (3D LPE),^50^ (3) a surface oxygen vacancies-engineered BiOCl-based photocatalytic solar interfacial evaporator (BOC/PDA/MF),^15^ and (4) a g-C_3_N_4_/MoS_2_ based floating solar still (CM-FSS).^51^ As depicted in Fig. 5a, GPA exhibited significant environmental benefits, and displayed relatively small impact on 12 environmental factors. Specifically, Fig. S23 shows that GPA had the lowest environmental impact across all categories amongst the four systems evaluated, with global warming at 6%, marine eutrophication at 1%, human carcinogenic toxicity at 1%, fine particulate matter formation at 5% and water consumption at 3%. These low carbon benefits are intrinsically linked to the simple material composition and fabrication process of GPA.

The CO_2_ emission distribution for each production stage of GPA is presented in Fig. S24. Freeze drying accounted for 64.69% of total emissions and was identified as the dominant contributor, attributed to the refrigeration and vacuum conditions required. Cross linking accounted for 1.81%, and had little impact on total emissions because of its low energy and material consumption. In terms of carbon emission reduction, alternative technologies should be explored, and the process parameters of freeze drying should be optimized. Furthermore, the material preparation costs of GPA were compared with those of the other four purification systems, highlighting the higher cost-effectiveness of GPA (Fig. 5b). Given its superior performance, low environmental impact and high cost-effectiveness, GPA is a promising candidate for next-generation carbon capture systems targeting amine-rich wastewater (Fig. 5c). Based on the strong hydrogen bond-mediated capture strategy, it can effectively prevent the co-evaporation of MEA and produce clean water for reuse in the wash water unit, thereby facilitating material balance control.

## 4. Conclusions

Solar-driven purification offers great promise for addressing the problem of carbon capture and wastewater discharge, but alkanolamine co-evaporation remains a key challenge, risking secondary atmospheric release of volatile amines. This study presents a novel approach to suppressing alkanolamine volatilization by developing a bifunctional graphene oxide-polyvinyl alcohol aerogel (GPA) that leverages selective hydrogen bonding. DFT and MD simulations confirm that the synergistic hydrogen bonding between –OH in PVA and –COOH in GO selectively immobilizes MEA without hindering water diffusion, as exemplified by the long GO-MEA hydrogen bond lifetime of 6.1 ps. Consequently, GPA achieves 99.5% MEA removal and an evaporation rate of 1.36 kg m^−2^ h^−1^ under 1 sun, with >95% removal sustained over 20 days and under extreme pH conditions. Life-cycle assessment further confirms that GPA contributes only 6% to global warming, the lowest among comparable systems. By integrating molecular-level selective retention with solar evaporation, this strategy offers a sustainable route to mitigate alcohol-amine emissions and opens a new route for integrating solar purification into next-generation carbon capture systems. In addition, the scalable fabrication and high-concentration MEA removal performance of GPA already indicated its practical potential, while future pilot-scale validation under actual flue-gas conditions will be essential to facilitate large-scale deployment.

## Author contributions

Yumeng Xiao: investigation, methodology, visualization, writing – original draft, formal analysis. Xinru Yang: investigation, formal analysis, visualization. Pengyang Liu: visualization. Xiangqian Song: visualization. Ruimin Zhao: formal analysis. Lidong Wang: conceptualization, funding acquisition, writing – review & editing. Meng Li: funding acquisition, writing – review & editing, data curation. Tony D. James: writing – review & editing, supervision.

## Conflicts of interest

There are no conflicts of interest to declare.

## Data Availability

The data supporting this article have been included as part of the supplementary information (SI). Supplementary information: experimental procedures, characterization data, supplementary figures and tables. See DOI: https://doi.org/10.1039/d6sc05691a.
